# Preclinical evaluation of a novel antibiotic-eluting BioEnvelope for CIED infection prevention

**DOI:** 10.3389/fddev.2024.1441956

**Published:** 2024-09-10

**Authors:** Zerelda Esquer Garrigos, John N. Catanzaro, Daniel Deegan, Ji Zhang, M. Rizwan Sohail

**Affiliations:** ^1^ Division of Infectious Diseases, Department of Medicine, University of Mississippi Medical Center, Jackson, MS, United States; ^2^ Division of Infectious Diseases, Department of Medicine, Mayo Clinic, Rochester, MN, United States; ^3^ East Carolina Heart Institute at ECU Health Medical Center, Greenville, IL, United States; ^4^ Elutia Inc, Silver Spring, MD, United States; ^5^ Section of Infectious Diseases, Department of Medicine, Baylor College of Medicine, Houston, TX, United States

**Keywords:** cardiac implantable device envelope, CIED, antibacterial envelope, biologic envelope, biomaterial-supported drug delivery, rifampin, minocycline

## Abstract

The risk of infection remains a significant concern with cardiovascular implantable electronic devices, necessitating the development of new strategies. This study explores the efficacy of a novel antibiotic-eluting biologic envelope designed to mitigate infection risk through localized antibiotic delivery while preserving the regenerative properties of biological matrix. Antibiotics, rifampin and minocycline, are released through polymer discs, ensuring extended drug release. Utilizing an established model of infection in a New Zealand White rabbit, the study assessed performance against Gram-positive bacterial strains, including common pathogens such as *Staphylococcus aureus* and *S. epidermidis* associated with CIED infections, and Gram-negative bacterial strains. Results demonstrated strong antibacterial activity, achieving complete eradication of bacterial colonies and greater than 6-log reductions in colonization for all strains. Pharmacokinetic analysis revealed sustained local antibiotic concentrations at the implantation site for up to 14 days, with minimal systemic exposure, demonstrating the advantages of localized drug delivery. Health outcomes in the antibiotic bioenvelope group were significantly improved, with no signs of infection or abnormal body temperatures, in contrast to the control group. Macroscopic examinations post-necropsy confirmed the absence of infection at the implantation sites of animals receiving the antibiotic bioenvelope. The combination of localized antibiotic delivery in a regenerative matrix positions the antibiotic bioenvelope as a promising solution for preventing CIED-related infections.

## 1 Introduction

Biomaterials are integral to modern medical devices, particularly in the field of cardiovascular diseases. They are utilized in various forms such as scaffolds, fibers, and coatings, offering mechanical support, tissue regeneration, and drug delivery capabilities. Cardiovascular implantable electronic devices (CIEDs) play a crucial role in managing various heart conditions but also pose a risk of complications, including device migration, erosion, and infection. CIED-related infections are associated with significant morbidity, mortality, and financial cost (Greenspon et al., 2018; Sohail et al., 2016). Effective strategies to prevent infections are essential to improve patient care and mitigate the burden on the healthcare system (Baddour et al., 2010; Blomström-Lundqvist et al., 2020).

Infection rates are as high as 4% in patients who undergo CIED procedures (Wilkoff et al., 2020). Bacterial strains are commonly introduced through the patient’s skin or the surgical environment (Hussein et al., 2016). Gram-positive bacterial species account for approximately 90% of these CIED infections including 29% by *Staphylococcus epidermidis* and up to 15% by methicillin-resistant *Staphylococcus aureus* (Sohail et al., 2007; Hussein et al., 2016). Gram-negative bacterial strains such as *Acinetobacter baumannii* and *Haemophilus influenzae* account for less than 2% of CIED infections but are exceedingly problematic due to their high resistance to antimicrobial measures (Sohail et al., 2007). The resistance of these bacterial strains makes finding the right antibiotic cocktail vital to infection prevention.

The introduction of microorganisms onto tissues surrounding the surgical site and/or the device surface can lead to bacterial colonization. Bacterial colonization is initiated during the initial implantation of CIEDs or after the procedure when a poor wound healing response in the subcutaneous pocket can lead to device erosion and bacterial infiltration. Colonization of these devices does not assure clinical infection, but all surgical-site infections derive from bacterial colonization. This colonization can result in formation of antimicrobial-resistant biofilms or eventual infection when disruptions in the host’s immune response allow for bacterial overgrowth (Büttner et al., 2015; Gristina et al., 1988). Studies have demonstrated the persistence of bacteria in the pocket post-implantation, even in the absence of acute clinical infection (Kleemann et al., 2010) or in asymptomatic patients undergoing reoperation (Chu et al., 2014). These bacterial biofilms may be released upon reoperation and increase the risk of subsequent infection. These findings underscore the importance of aggressively eliminating or minimizing bacterial colonization post-implantation to reduce the risk of infection over the CIED’s lifetime.

Poor stability or healing around an implant increases the chance of device erosion and exposure to pathogens, while an excessive fibrotic response can trap bacteria in hypovascular capsular tissue of the surgical pocket further increasing infection risk during reoperations. Device envelopes have been developed that address challenges related to stabilizing CIEDs in the pocket and reducing bacterial colonization. One such envelope (CanGaroo^®^ Envelope, Elutia Inc., Silver Spring, MD) is composed of biologic extracellular matrix (ECM) that provides a conforming material that secures the CIED while supporting tissue integration and vascularization. This biocompatible material mitigates chronic inflammation and fibrotic encapsulation of the device (Brown et al., 2012; Deegan et al., 2022). While a healthy, vascularized pocket lessens the chance of late-stage infection, local delivery of antibiotics at the CIED-host interface is a rational approach to further reduce the overall infection risk. This can be achieved through the addition of antibiotics to the device envelopes. The antibiotic envelope (TYRX™, Medtronic, Inc., Minneapolis, MN), a synthetic polymer mesh coated with rifampin and minocycline, has been shown to reduce the incidence of CIED pocket infections in high-risk patients (Tarakji et al., 2019). This concept builds on the successful use of drug-coated devices in other medical applications, such as central venous catheters, which were among the earliest devices that were coated with antibiotics and shown to lower infection rates (Chaftari et al., 2014).

There is a growing recognition of the importance of combining the benefits of local drug delivery with the regenerative potential of biologic extracellular matrix (ECM). A next-generation antibiotic-eluting biologic envelope has been developed by adding rifampin and minocycline while preserving the desirable qualities of the regenerative biomaterial ECM. The efficacy of rifampin and minocycline in reducing surgical site infections is well-documented and reduces the rate of infections associated with medical devices (Chemaly et al., 2010). The broad-spectrum coverage provided by this combination includes Gram-positive species found in most CIED infections (e.g., *Staphylococcus epidermidis* and methicillin-resistant *S. aureus*), as well as Gram-negative species, some of which the Center for Disease Control and Prevention (CDC) has reported as increasingly drug-resistant (Hussein et al., 2016; Sohail et al., 2007; Tarakji et al., 2010).

To preserve the tissue integration and regenerating properties of the ECM biomaterial, a novel drug delivery system was developed to minimize any physical or chemical impact on the ECM. Unlike traditional drug coating or impregnation technology, a stand-alone drug-eluting disc made from bioabsorbable polymer was incorporated into the biologic envelope, enabling antibiotic delivery to protect the entire device. This approach is elegant in its simplicity, leading to minimal impact on the important ECM functionalities, such as surface properties and porosity, which are critical to promote cell infiltration, proliferation, and healthy tissue regeneration. Furthermore, the drug-eluting disc can be independently formulated for different dosages and elution profiles. It is a versatile drug-eluting platform that can be easily incorporated into different biomaterials or medical devices to prevent infections. This study evaluated the antibacterial efficacy of this antibiotic bioenvelope *in vivo* using an established preclinical animal model of CIED infection. A range of organisms were tested to ensure that the envelope’s effectiveness spanned the spectrum of potential pathogens, including Gram-positive organisms which are commonly associated with CIED-related infections.

## 2 Methods

### 2.1 Antibiotic-eluting bioenvelope

**FIGURE 1:**
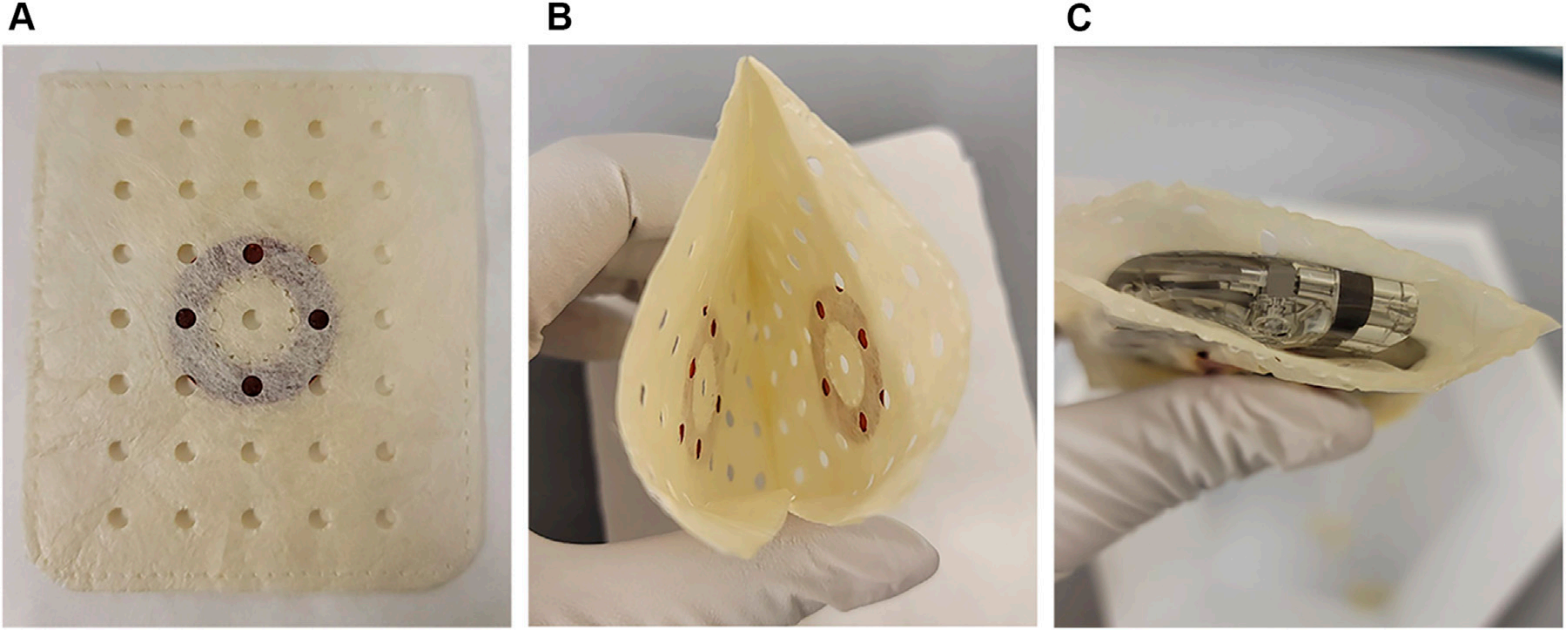
The antibiotic bioenvelope. **(A)** Top view of the antibiotic bioenvelope, illustrating a drug-eluting polymer disc containing rifampin and minocycline, immobilized between multilaminate extracellular matrix (ECM) sheets. The polymer composition of the disc is Poly (D,L-lactide-co-glycolide) (PLGA). **(B)** The configuration of the drug discs on the opposite surfaces of the envelope. **(C)** The antibiotic bioenvelope is designed for the insertion of a cardiovascular implantable electronic device (CIED) or a neurostimulation device. Images depict the antibiotic bioenvelope in its non-hydrated state.

The device under study is an antibiotic-eluting bioenvelope comprising decellularized, non-crosslinked extracellular matrix (ECM) with drug-eluting discs (EluPro™, Elutia, Silver Spring MD), which is designed to secure and stabilize a CIED upon implantation, as shown in Figure 1. The ECM component of the bioenvelope was derived from porcine small intestinal submucosa (SIS) and constructed with multilaminate sheets, perforated to allow for drainage of exudate. The drug-eluting polymer discs were fabricated using a solvent casting method. This involves combining the resorbable copolymer poly (lactide-co-glycolide) (PLGA) with rifampin and minocycline, followed by molding and drying into a ring shape. Given the different solubilities of rifampin (2.5 mg/mL) and minocycline (50 mg/mL) in PBS, the discs were engineered with a specific PLGA formulation that enables controlled drug release through both diffusion and degradation mechanisms over a period of at least 7 days. PLGA is a hydrophobic polymer, which helps control the rate of water penetration and subsequently the hydrolysis process, affecting both the degradation rate and drug release profile. Physiochemical analysis revealed that the discs have a glass transition temperature (Tg) of 47°C, ensuring that the drug-eluting discs maintain their physical state *in vivo*. The antibiotic bioenvelope has a minimal nominal drug per surface area of 95 μg/cm^2^ for rifampin and 85 μg/cm^2^ for minocycline. The device was assessed for biocompatibility following ISO 10993 standards.

### 2.2 Animal experiments

All animal studies conformed to the Guide for the Care and Use of Laboratory Animals, and the surgical procedures and animal care were conducted at independent research organizations in compliance to Good Laboratory Practice. The Institutional Animal Care and Use Committee at WuXi AppTec (Saint Paul, MN) approved all animal study protocols prior to study initiation.

### 2.3 Bacterial dosing study

A dosing study was conducted to determine the appropriate inoculum dosage for the subsequent efficacy study. Experiments were conducted using the established New Zealand White rabbit cardiac device pocket colonization model (Hansen et al., 2009; Sohail et al., 2020). A total of six bacterial species were investigated, including three Gram-positive (methicillin-resistant *S. aureus* [MRSA, ATCC 33591], *S. aureus* [ATCC 29213], *S. epidermidis* [ATCC 35984]) and three Gram-negative (*Escherichia coli* [ATCC 25922], *Acinetobacter baumannii* [ATCC 19606], and *Haemophilus influenzae* [ATCC 53782]).

For each bacterial group, nine New Zealand White rabbits were evaluated, with three animals tested per inoculum dosages. Three concentrations spanning three logarithmic orders of magnitude were tested for each bacterial strain (termed low, medium, and high). For example, 1.0 × 10^6±0.5^ CFU/mL (low), 1.0 × 10^7±0.5^ CFU/mL (medium), and 1.0 × 10^8±0.5^ CFU/mL (high) concentrations of *S. epidermidis* were tested. The concentrations of the inoculum tested were determined based on the virulence and characteristics of the specific bacterial strain under investigation. The objective of the dosing study was to determine the strains and appropriate inoculum dosages that would allow demonstration of at least a 6-log reduction in bacterial colonization in the efficacy study.

The animals underwent implantation of bilateral dorsal subcutaneous devices, creating separate pockets through a single skin incision. The implants consisted of a nonfunctioning, sterilized pacemaker (Biotronik, Inc., Lake Oswego, OR) encased by a non-drug version of the envelope (NDE). The NDE samples were hydrated in 20 mL of sterile saline for 2 min prior to implantation.

Following the closure of the implant pockets, 1 mL of the target bacterial inoculum concentration was delivered into each implant site through a catheter tunnelled subcutaneously into the pocket. For example, for the high inoculum concentration of *S. epidermidis*, 1 mL would inoculate the pocket with 1.0 × 10^8±0.5^ CFU. This was followed by a 1 mL saline flush for complete delivery of the inoculum. A microchip was placed subcutaneously for body temperature monitoring.

After surgery, animals were monitored twice daily for general health and body temperature for 1 week. Animals were considered for unscheduled euthanasia if they experienced weight loss exceeding 20% of their initial weight with signs of illness or distress, prolonged anorexia lasting more than 3 days, or health problems unresponsive to treatment. After termination or early death, each implant site was aseptically exposed and examined for macroscopic findings.

The optimal inoculum dose for the subsequent efficacy study was determined based on the bacterial concentration of each strain that established a consistent, non-lethal, localized infection in the subcutaneous pocket of the rabbit model. The dosing study aimed to ensure consistent dosing between control and test animals. These data helped guide the design of the efficacy study, considering tolerability of the specific bacterial strains and inoculum levels.

### 2.4 *In vivo* antibacterial efficacy studies

To analyze the antibacterial performance of the antibiotic bioenvelope *in vivo*, experiments were conducted using the same New Zealand White rabbit cardiac device pocket colonization model described above for the dosing studies. The test group was implanted with the antibiotic bioenvelope and inoculated with individual bacterial strains. The control group used a non-drug envelope (NDE) and was inoculated with individual bacteria strains. Five animals were included in each group. All envelopes were implanted containing a non-functioning pacemaker (Biotronik, Lake Oswego, OR). The device utilized represented the lowest nominal antibiotic concentration of the range of sizes, as measured by the amount of antibiotic per envelope area.

Bacterial species and their respective inoculation concentrations used in the efficacy study were determined from the highest survivable level in the dosing study that ensured a consistent, non-lethal, localized infection (Table 1).

**TABLE 1:** Summary of inoculum doses for bacterial strains based on *in vivo* dosing studies.

Bacterial strain	Gram stain	Inoculum concentration (CFU/mL)
Methicillin-resistant *Staphylococcus aureus* (MRSA) ATCC 33591	Positive	1.0 × 10^6^
*Staphylococcus epidermidis* ATCC 35984	Positive	1.0 × 10^8^
*Acinetobacter baumannii* ATCC 19606	Negative	1.0 × 10^6^
*Haemophilus influenzae* ATCC 53782	Negative	1.0 × 10^6^

The surgical procedure was similar to that of the dosing study described above. Implant pockets were created dorsally, one on each side of the midline, and each study animal received two implants in a bilateral configuration. Envelopes were hydrated in sterile saline prior to implantation. After implant, 1 mL of the appropriate inoculum concentration was delivered into each individual subcutaneous pocket through a catheter tunneled subcutaneously into the pocket via a separate incision, followed by a 1 mL saline flush to ensure complete delivery. A microchip was placed subcutaneously for body temperature monitoring. Animals were monitored twice daily for general health observations and temperature.

After 7 days, surviving animals were humanely euthanized, and a gross necropsy analysis was performed. The implants and surrounding tissue were aseptically explanted and separately assessed for bacterial recovery. Each envelope was placed in an individual container with sonication buffer (Dey-Engley broth with 5% Tween 80). Tissue samples were collected from the surrounding tissue pocket and minced in a separate container with sonication buffer. Sonication solutions were plated onto non-selective TSA plates to determine bacterial recovery.

### 2.5 *In vivo* drug content of the antibiotic bioenvelope

A pharmacokinetics (PK) study was conducted to evaluate the *in vivo* antibiotic elution profile and systemic exposure levels of rifampin and minocycline following implantation of the antibiotic bioenvelope. The study aimed to measure the drug concentrations in serum and drug levels of the device *in vivo*. A total of 24 New Zealand White rabbits were included the study, with each animal receiving an implantation of an antibiotic bioenvelope in the dorsal subcutaneous tissue. Each envelope contained a non-functioning CIED, consistent with previous studies. The animals were divided into six groups, with four animals in each group, and were euthanized at six time points (2 h, 1, 3, 5, 7, and 14 days) post-implantation. This study design allowed for a comprehensive assessment over a 14-day period, to determine antibiotic coverage through the target duration of 7 days.

Serum samples were collected at various time points (15 and 30 min; 1, 2, 6, 24, 36, 48, and 72 h; and 7 and 14 days) for analysis. After the predefined survival periods, animals were euthanized, and the antibiotic bioenvelope devices were explanted for quantitative drug analysis. Serum and explant samples were stored frozen (−80°C) until analytical assessment.

For the quantification of rifampin and minocycline, serum samples were analyzed using a validated liquid chromatography/mass spectrometry (LC/MS) method. Disc extractions were performed in methanol, filtered, and transferred for analysis. Selectivity was ensured using blank serum samples.

Explanted devices were analyzed using a validated high performance liquid chromatography with ultraviolet detection (HPLC-UV) method. The amount of drug eluted was determined by subtracting the residual antibiotic for each device from the respective label claim.

### 2.6 Data analysis

Data sets were processed in Excel (Microsoft) and GraphPad (GraphPad Software, Boston, MA, United States). The biological replicates’ values of CFU counts or percent label claim of drug released were averaged and then reported as mean ± standard error (if applicable). Mann-Whitney U tests were used to calculate statistical differences between the antibiotic bioenvelope group and initial dose CFUs for each bacterial strain tested. Fisher’s Exact tests were done to calculate statistical differences for clinical outcomes between the antibiotic bioenvelope and nondrug control groups. Analysis of variance (ANOVA, 1-factor) with Tukey-Kramer was used for comparison between the clinical trough levels *versus* serum levels of rifampin and minocycline over time. A *p*-value <0.05 was deemed significantly different for all tests.

## 3 Results

### 3.1 Inoculum doses optimized for each bacterial strain

The dosing study was completed to establish the optimal bacterial concentration of each strain for inducing a consistent, non-lethal infection in the subcutaneous implant model using a non-drug envelope. The study established target inoculum concentrations for subsequent efficacy evaluations, as summarized in Table 1. For Gram-positive bacteria, escalating doses of *S. epidermidis* did not elicit death in any of the nine animals across each dosing group during the 7-day study. Based on stringent infection acceptance criteria and in-life health observations, a target concentration of 10^8±0.5^ CFU/mL was deemed suitable for the subsequent efficacy study. In the case of MRSA, all administered doses resulted in a minimum of one early death, necessitating the selection of the lowest target concentration of 10^6±0.5^ CFU/mL for the efficacy study. *Staphylococcus aureus* inoculations led to mortality across all three dose levels. As the target dose of at least 10^6^ could not be achieved with this virulent strain, it was not tested in the efficacy study.

For Gram-negative bacterial strains, none of the 3 *A. baumannii* or *H. influenzae* doses elicited any animal deaths in studies with the non-drug envelope. Therefore, the dose concentration of 10^6±0.5^ CFU/mL for both strains was selected to establish the most consistent infection for the efficacy studies. In contrast, the high and medium doses of *E. coli* led to mortality, while the low dose established a consistent non–lethal infection at the implant site. However, this did not meet the objective of the minimum target dose and was not utilized in the efficacy study.

### 3.2 Use of antibiotic bioenvelope improves preclinical health outcomes

During the 7-day in-life period, animals were closely monitored for any health complications, with necessary interventions provided by veterinarians as required (Table 2). Following the initial surgery, all test group animals who received the antibiotic bioenvelope appeared healthy throughout the study. None of these animals exhibited abnormal body temperatures or required veterinary intervention. In contrast, in the nondrug control group, body temperature monitoring showed three out of 20 animals (3/5 *H. influenzae* inoculated rabbits) experienced hyperthermia (>40°C) requiring veterinary attention. Additionally, 7 out of 20 animals required supportive care, typically initiated between days 3–5. Health observations of this group were consistent with the presence of an active, localized, bacterial infection. One animal in the *S. epidermidis* group developed a hematoma. Fisher’s exact test demonstrated statistically significant differences (*p* < .05) between the antibiotic bioenvelope and nondrug control groups for number of animals requiring supportive care in the individual MRSA inoculum arm as well as in the total animals across all bacterial strains.

**TABLE 2:** Summary of in-life health observations by treatment group for each bacterial strain.

Bacterial strain	Febrile/Hyperthermia		Required supportive care		Early termination/Premature death	
	Antibiotic bioenvelope	Nondrug control	Antibiotic bioenvelope	Nondrug control	Antibiotic bioenvelope	Nondrug control
MRSA ATCC 33591	0/5	0/5	0/5	5/5 by day 3–5	0/5	0/5
*Staphylococcus epidermidis* ATCC 35984	0/5	0/5	0/5	0/5	0/5	0/5
*Acinetobacter baumannii* ATCC 19606	0/5	0/5	0/5	0/5	0/5	0/5
*Haemophilus influenzae* ATCC 53782	0/5	3/5	0/5	2/5 by day 3	0/5	1/5
**Total**	**0/20**	**3/20**	**0/20**	**7/20**	**0/20**	**1/20**

Bold values = Total animals across all bacterial strains.

Furthermore, there were no early terminations or premature deaths in the antibiotic bioenvelope group, and there was one unscheduled death in the control group (1/5 H. influenzae inoculated rabbits).

### 3.3 The *in vivo* use of antibiotic bioenvelope results in bacterial eradication

**FIGURE 2:**
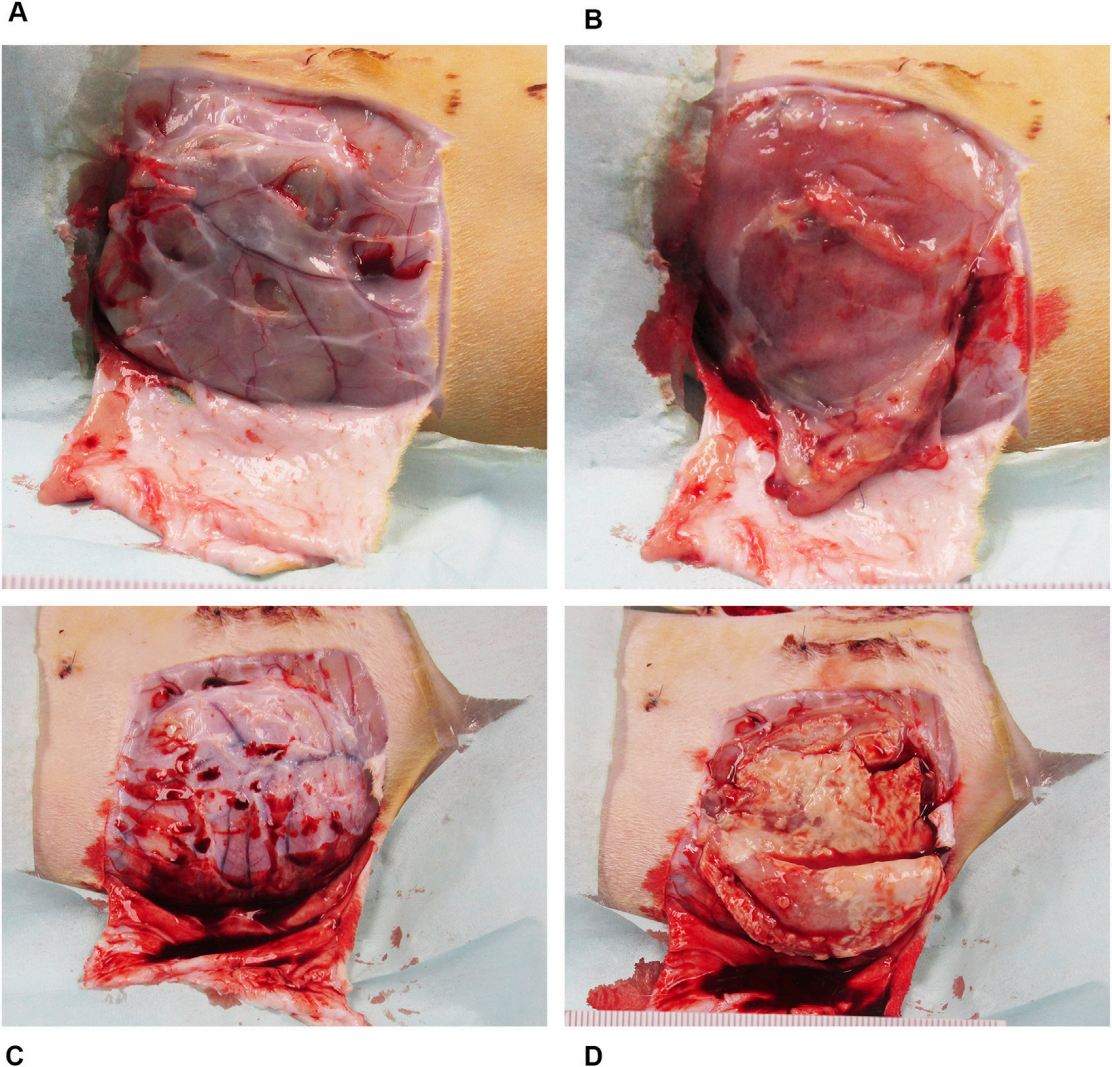
Representative necropsy images of the device pocket at sacrifice from MRSA bacterial challenge group. **(A)** The surgical site with the antibiotic bioenvelope exposed: Image of the soft tissue covering the implant site with skin resected. **(B)** Subcutaneous pocket tissue after removal of the implanted antibiotic bioenvelope, showing a normal, healthy pocket. **(C)** Surgical site with soft tissue covering the implant site of a control sample with the skin resected, providing a comparative view of the control group’s surgical outcome. **(D)** Subcutaneous pocket after removal of the implanted control device, highlighting pathological changes associated with the infectious process.

At the end of the study, necropsy revealed that sites implanted with the antibiotic bioenvelope appeared macroscopically normal, while control animals showed signs of infection. Figure 2 shows representative photographs at necropsy of both groups. In the antibiotic bioenvelope group, no white matter or thickened tissue was observed in the implant sites. In contrast, control animals exhibited white matter, fluid, and thickened tissue in both the left and right pockets, indicative of inflammatory processes and early signs of infection (Table 3). These responses confirm the validity of this infection model by demonstrating that the animal immune system alone was unable to overcome the bacterial challenge.

**TABLE 3:** Summary of gross necropsy observations of the surgical site by treatment group.

Bacterial strain	Observations	
	Antibiotic bioenvelope	Nondrug control
MRSA ATCC 33591	10/10 macroscopically normal	10/10 Presence of white matter, fluid, and thickened tissue
*Staphylococcus epidermidis* ATCC 35984	10/10 macroscopically normal, one hematoma noted	10/10 Presence of white matter, fluid, and thickened tissue
*Acinetobacter baumannii* ATCC 19606	10/10 macroscopically normal	10/10 Presence of white matter, fluid, and thickened tissue
*Haemophilus influenzae* ATCC 53782	10/10 macroscopically normal	10/10 Presence of white matter and thickened tissue
Summary	40/40 macroscopically normal	40/40 white matter, fluid, and thickening consistent with infection

Total animals across all bacterial strains.

The antibiotic bioenvelope displayed strong antibacterial effectiveness against all Gram-positive and Gram-negative species tested. Test envelopes demonstrated complete bacterial eradication and achieved greater than 6-log reductions compared to the inoculum for MRSA, *S. epidermidis*, *A. baumannii*, and *H. influenzae* (Table 4). Bacterial recovery counts showed no viable bacteria in the test envelopes, indicating the high efficacy of the antibiotic bioenvelope. One test sample that showed contamination in the extract samples was omitted from the average result calculation. Mann-Whitney U tests yielded a *p*-value well below the significance threshold of 0.05, indicating that the reduction in CFUs compared to the inoculum dose for each bacterial strain is highly statistically significant.

**TABLE 4:** Antibiotic bioenvelope performance against bacterial species *in vivo*.

Organism	Gram stain	Inoculum	Inoculum recovery (mean CFU) n = 10	Antibacterial efficacy^a^	Reduction of bacterial colonization
*S. epidermidis*	positive	10^8^	CIED: 0	>8-log	Complete kill
			Host Tissue: 0		
			Antibiotic bioenvelope: 0		
MRSA	positive	10^6^	CIED: 0	>6-log	Complete kill
			Host Tissue: 0		
			Antibiotic Bioenvelope: 0		
*A. baumannii*	negative	10^6^	CIED: 0	>6-log	Complete kill
			Host Tissue: 0		
			Antibiotic Bioenvelope: 0		
*H. influenzae*	negative	10^6^	CIED: 0	>6-log	Complete kill
			Host Tissue: 0		
			Antibiotic Bioenvelope: 0		

^a^ Log_10_ reduction of the average recovered organism counts from the CIED, implants, and surrounding tissue of surgical pockets of the test envelope group versus the initial inoculum for each bacterial strain.

### 3.4 Antibiotic bioenvelope demonstrates sustained local drug release with minimal systemic exposure

**FIGURE 3:**
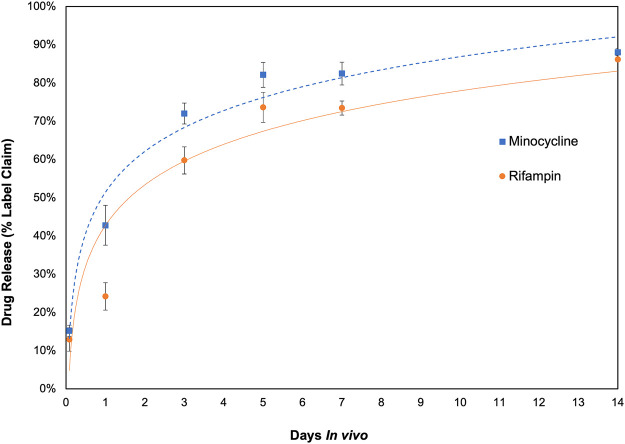
*In vivo* drug release profile of the antibiotic bioenvelope in a rabbit model. The graph depicts the cumulative amount of rifampin and minocycline eluted, indicating extended drug release over a 14-day period. Error bars represent the standard error (n = 4 per time point).

The investigation of *in vivo* drug elution and pharmacokinetics of rifampin and minocycline demonstrated significant differences between local and systemic drug levels in the rabbit model, with local levels orders of magnitude greater than systemic levels. Measurements of drug levels in the antibiotic bioenvelope were taken at 2 h, and at 1, 3, 5, 7, and 14 days after device explantation, showing antibiotic release throughout the 2-week duration. Figure 3 illustrates the cumulative drug release over time, indicating a more rapid release in the early days, with over half of the drug eluted by day 3, and then a tapering of release for the remainder of the study. The total drug released through 14 days averaged 9.0 ± 0.12 mg for rifampin and 8.2 ± 0.17 mg for minocycline. Approximately 10% of the drug content remained at the final study time point of 14 days.

**FIGURE 4:**
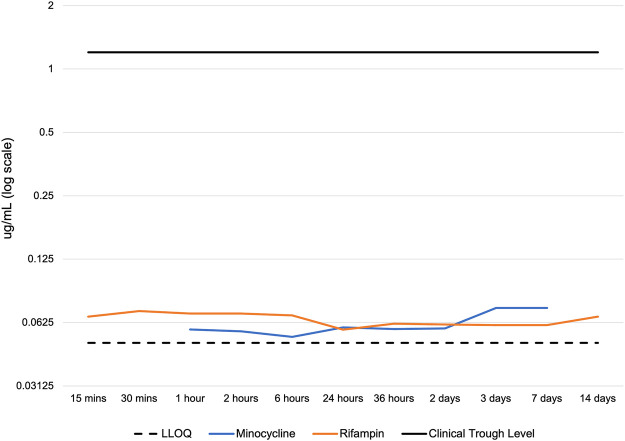
Serum concentration of rifampin and minocycline after device implantation. LLOQ: lower limit of quantitation of the LC-MS/MS. Clinical trough levels are approximately 1.2 - 2 ug/mL for rifampin and minocycline, which represent the lowest serum drug concentration observed in patients immediately before the next dose. The serum concentration of minocycline was below the LLOQ at 15 min, 30 min, and 14 days. The LLOQ was >0.046 μg/mL for minocycline and >0.049 μg/mL for rifampin.

Conversely, serum levels of rifampin and minocycline in serum were consistently low, with concentrations of both antibiotics below 0.08 μg/mL at all time points studied. These levels were well below the clinical trough concentration in humans after systemic administration of rifampin (peak level 17.4 μg/mL, trough 1.2 μg/mL) and minocycline (peak level, 4.18 μg/mL, trough 1–2 μg/mL) (Figure 4) and significantly different when compared by ANOVA with Tukey-Kramer tests over the 14-day time period. Meaningful drug levels were observed to be limited to the local site of delivery, and accordingly, the drugs had negligible systemic exposure. Considering the size difference and total blood volume (at least 20-fold greater in humans than rabbits), serum levels of both drugs would be expected to be below detectable limits in clinical use.

## 4 Discussion

### 4.1 Efficacy of antibiotic bioenvelope

This study evaluated the efficacy of a novel drug-eluting biologic envelope designed to prevent CIED and related device infections. This antibiotic bioenvelope incorporates resorbable PLGA discs impregnated with rifampin and minocycline, delivering antibiotics locally with minimal systemic exposure. Results demonstrated complete bacterial eradication and greater than 6-log reductions for both Gram-positive and Gram-negative bacterial strains tested. This effectiveness was consistent across multiple bacterial species commonly associated with CIED infections, such as *S*. *aureus* and *S. epidermidis*, underscoring the broad-spectrum coverage provided by the antibiotic combination of rifampin and minocycline. Achieving bacterial eradication translated into significant improvements in animal health. With extended-release capacity for at least 2 weeks, the device offers protection against the initiation of infections by eliminating bacterial colonization. This is especially critical in the early post-implantation period when the risk of infection is elevated.

### 4.2 Critical defense against initiation of infection

The potential for bacterial colonization on implanted biomaterials poses immediate and persistent infection risks, especially during subsequent interventions like device exchanges or upgrades (Harper et al., 2018; Subbiahdoss et al., 2009). The antibiotic bioenvelope effectively eliminates bacterial colonization across various tested bacterial species, as observed by *in vivo* studies in preclinical models. This effectiveness stems from its prolonged release of rifampin and minocycline over a period exceeding 1 week *in vivo*, effectively impeding biofilm formation. Proactive intervention with antibiotics is crucial, given that biofilms can serve as a nidus for infection, remaining asymptomatic for a time yet posing a latent risk during future surgical interventions (Harper et al., 2018; Nagpal et al., 2012). By preventing bacterial colonization, the biologic envelope serves as a key defense against CIED-related infections.

The efficacy demonstrated in this study was particularly compelling given the use of an established preclinical model that provides robust evidence of the biologic envelope’s effectiveness in a clinically relevant context. The observed reductions in bacterial load underscore the capacity of the envelope to disrupt the early stages of infection establishment, thereby preventing the progression to clinically significant infections. Furthermore, the improvement in microbial counts was mirrored by enhancements in animal health and signs of infection. This alignment between quantitative microbial results and animal symptomatology demonstrates the validity of the model and clinical relevance of the findings.

### 4.3 Advantages of local drug delivery

The local drug delivery system demonstrated in this study offers several advantages over systemic drug administration. The *in vivo* drug content and pharmacokinetic data showed that the device could deliver therapeutic antibiotics locally for at least 14 days with minimal systemic drug exposure. This localized delivery approach allows for higher drug concentrations at the target site, which is particularly beneficial in combating infections associated with implanted medical devices (Chaftari et al., 2014; Hansen et al., 2010). By delivering antibiotics directly to the site of infection, the risk of systemic side effects is reduced, as the drugs are not circulating throughout the entire body at high concentrations. Additionally, the sustained release of antibiotics over a period of 2 weeks minimizes the need for frequent dosing, improving patient compliance and reducing the burden of care. This targeted delivery system also helps prevent the development of antibiotic resistance, as the local concentration of antibiotics remains effective against bacteria without the need for higher systemic doses. Overall, the local drug delivery system presented in this study offers a promising approach for the treatment and prevention of infections related to implantable devices.

### 4.4 Benefits of biologic ECM

The antibiotic bioenvelope, composed of decellularized, non-crosslinked ECM, fosters tissue regeneration by creating a supportive environment for tissue regeneration. Additionally, the ECM naturally modulates the immune response to mitigate inflammation and promote tissue vascularization and integration, essential for positive outcomes post-implantation (Deegan et al., 2022; Wolf et al., 2014). Moreover, the flexibility and ease of handling of biologic ECM can facilitate surgical implantation, improving the overall functionality of the device. The capacity of biologic ECM to integrate into native tissue is also pivotal for the device’s long-term performance. This material has demonstrated the ability to induce remodeling into vascularized and cellularized tissue, potentially inhibiting excessive fibrotic adhesions around the CIED post-implantation, thereby reducing the persistence of trapped bacteria within a capsule (Brown and Badylak, 2014; Dziki et al., 2017). Clinical reports indicate that patients who received ECM envelopes during their initial surgery had easier reoperations with fewer lead adhesions and less capsulectomy during subsequent procedures compared to those who did not receive an envelope or received the non-biologic envelope (Catanzaro et al., 2023). Furthermore, a recent case study presented histologic evidence of the use of a biologic envelope within an established dense fibrotic capsule to rejuvenate the device pocket, resulting in regeneration of healthy, vascularized tissue around the device (Srivastava and Nayak, 2023). The biocompatibility and regenerative potential of biologic ECM make it an ideal material for use in drug-eluting medical devices.

### 4.5 Impact on reoperations and infection risk

Reoperations in CIED candidates, particularly young patients, pose a significant risk of infection due to potential bacterial colonization around the device. Fibrotic capsules, common around non-biologic materials, may contribute to bacterial persistence and growth, increasing the risk of infection (Harper et al., 2018; Kleemann et al., 2010). The antibiotic bioenvelope, derived from SIS-ECM, has been shown to facilitate reoperative procedures by preventing excessive fibrotic adhesions, potentially reducing bacterial persistence within capsules and lowering infection risk (Catanzaro et al., 2023). Clinical data support the potential benefits of biologic envelopes in reducing lead adhesions, facilitating reoperations, and requiring less capsulectomy compared to non-biologic envelopes (Catanzaro et al., 2023).

### 4.6 Conclusion and future directions

While local delivery of antibiotics offers significant advantages over systemic administration, certain considerations remain. Infections associated with devices such as CIEDs may not be limited to the implant site alone; systemic or distant infections could still pose a risk that local antibiotic therapy might not fully address. The prophylactic use of antibiotics may raise concerns about the risk of antibiotic resistance. Nonetheless, this risk may be lower with local delivery compared to systemic administration due to the limited exposure of bacteria to sub-therapeutic antibiotic levels. The acceptable risk from exposure to antibiotics is further supported by the fact that this is a single-use device, with a favorable risk-benefit profile. The use of an antibacterial envelope device reduces the risk of needing to remove a pacemaker or defibrillator and treat aggressively for infection with high-dose antibiotics. A review of clinical data on the combination of rifampin and minocycline found no evidence of antibiotic resistance (Reitzel et al., 2020). Ongoing monitoring for antibiotic resistance is important for the responsible use of antibiotics.

The study’s duration means that its evaluation period does not capture long-term performance and infection rates. These aspects would be appropriate to study clinically by collecting long-term follow-up data. Additionally, preclinical models may not fully replicate clinical symptoms and outcomes.

In conclusion, the antibiotic bioenvelope shows promise as a strategy for preventing CIED-related infections. Its ability to deliver therapeutic antibiotics locally while minimizing systemic exposure, along with its potential to facilitate tissue integration and reduce fibrotic capsule formation, highlights its clinical relevance and potential to improve patient outcomes. Future clinical studies will be useful to understand the benefits of an antibiotic bioenvelope not only in reducing infection but other complications such as erosion, discomfort, and overall patient experience throughout the lifetime of the device.

## Data Availability

The raw data supporting the conclusions of this article will be made available by the authors, without undue reservation.
